# Orbital Tumor As First Manifestation of Metastatic Renal Cell Carcinoma

**DOI:** 10.7759/cureus.16275

**Published:** 2021-07-09

**Authors:** Shu Yu Tan, Mae-Lyn Catherine Bastion, Safinaz Mohd Khialdin

**Affiliations:** 1 Ophthalmology, Universiti Kebangsaan Malaysia Medical Centre, Kuala Lumpur, MYS

**Keywords:** orbital metastasis, orbital tumor, renal cell carcinoma

## Abstract

Orbital metastasis from renal cell carcinoma (RCC) is uncommon. Orbital tumor, as the first presentation of RCC, is rare as the majority of orbital metastases occur after a confirmed diagnosis of primary cancer. We report a case of the metastatic orbital tumor as the first manifestation of RCC, which presented with painless left eye proptosis for two months' duration, associated with blurring of vision and diplopia. Otherwise, the systemic review was unremarkable. Examination showed left eye non-axial proptosis with a pulsatile, multilobulated mass over the left supraorbital area extending to the left frontal region, limited ocular motility, and impaired optic nerve functions. CT of the orbit showed a mass arising from the left frontal and greater wing of the left sphenoid bone, with infiltration to the left lateral rectus, left superior oblique, and lacrimal gland. Further systemic investigation with CT thorax, abdomen, and pelvis revealed left RCC with para-aortic nodes, lungs, and bone metastases. The patient was planned for palliative care.

## Introduction

Metastasis to the eye most commonly involves the uvea, especially the choroid, attributing to its vast blood supply [1]. Metastasis to the orbit is relatively infrequent. Orbital tumors in a cancer patient should be investigated for metastasis. The metastatic nature of an atypical orbital tumor should be considered even in those without a cancer history. We report a case of an orbital tumor presenting as the first manifestation of metastatic renal cell carcinoma (RCC).

## Case presentation

A 73-year-old female presented with two months history of painless left eye proptosis, progressive blurring of vision, and diplopia. The systemic review was unremarkable with no loss of appetite or weight, haematuria, flank pain, cough, or bony pain. She was a cigarette smoker of eight packs/year. Her daughter suffered from breast cancer.

**Figure 1:**
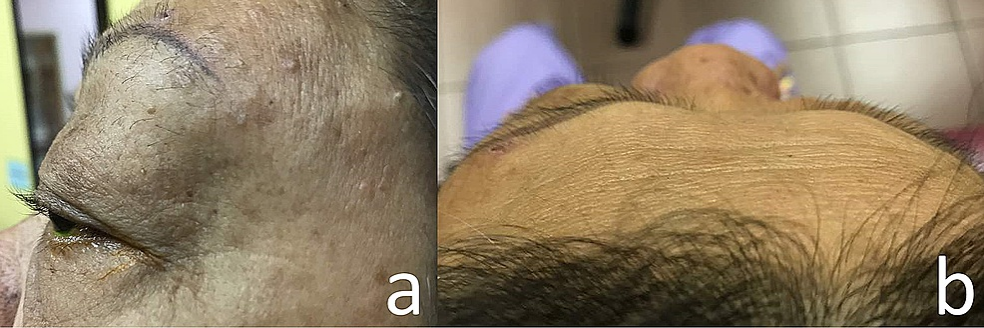
Non-axial proptosis of the left eye with a mass located over the left superotemporal part of orbit, extending to the left frontal region. 1a: Left sagittal view, 1b: Bird’s eye view.

**Figure 2:**
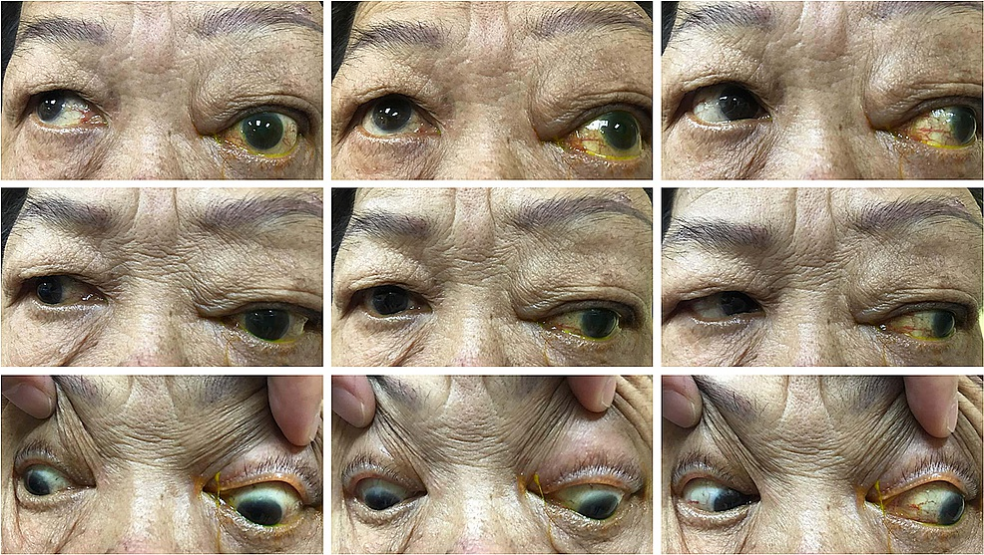
Restricted left eye elevation and adduction on motility examination.

Ophthalmic examination showed left eye inferotemporal dystopia with proptosis. A firm, non-tender, pulsatile multilobulated, firm mass measuring 6 cm by 6 cm was noted over the left superotemporal part of the orbit, extending to the supraorbital and left frontal region, with dilated superficial tortuous vessels, but no skin changes or bruit (Figure 1). Left eye elevation and adduction were restricted (Figure 2), with diplopia on all gazes except left and downgaze. Visual acuity was 6/12 in the right eye and 6/60 in the affected left eye. Left optic nerve function was impaired with the presence of a relative afferent pupillary defect. Anterior segment examination was unremarkable. Fundus examination showed a flat retina and no choroidal lesion. The left optic disc was pale with a cup-to-disc ratio of 0.7, whereas the right optic disc was pink with a cup-to-disc ratio of 0.3. Systemic examinations, which include abdomen examination, were unremarkable.

**Figure 3:**
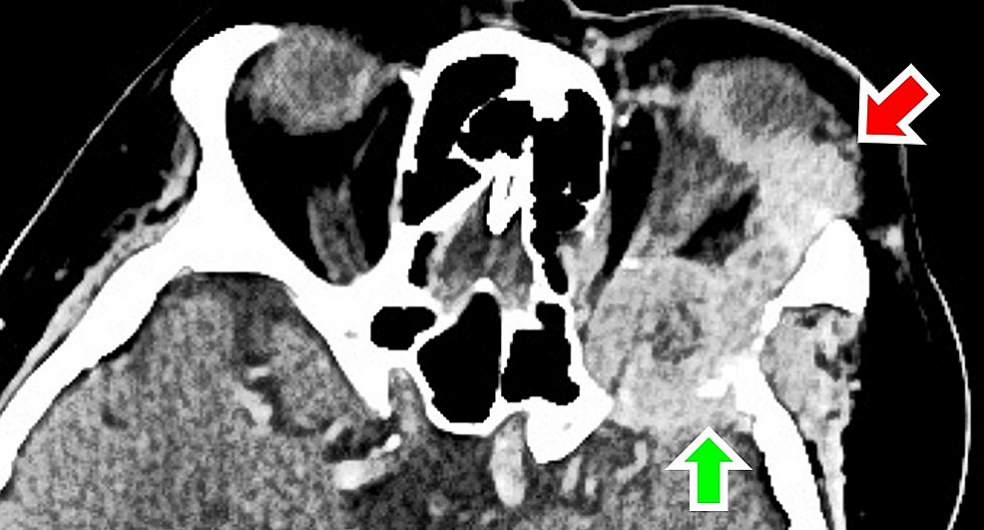
CT orbit showed an orbital mass (red arrow) with lytic destruction of the greater wing of the left sphenoid bone (green arrow).

**Figure 4:**
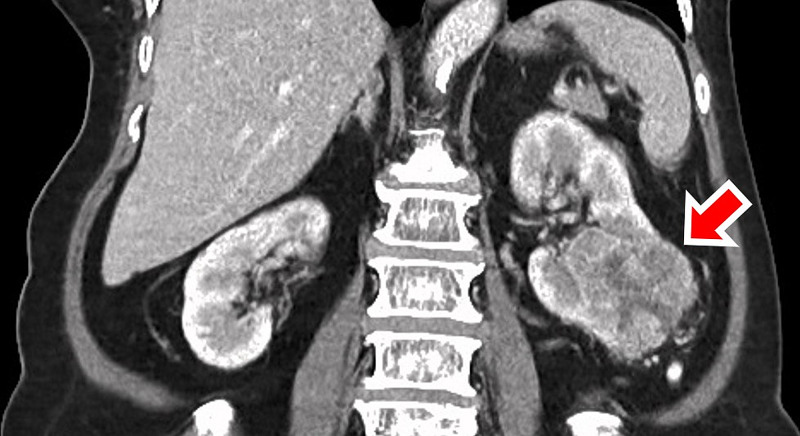
CT abdomen revealed left RCC (red arrow). RCC: Renal cell carcinoma.

CT of the orbit showed a mass arising from the left greater wing of the sphenoid bone, measuring 6.4 cm × 5.7 cm × 6.6 cm, extending to left frontal sinus and intraconal space, infiltrating lateral rectus, superior oblique muscles, and lacrimal gland (Figure 3). Full blood count and renal profile were normal. Tumor markers, including carcinoembryonic antigen, CA 125, CA 19-9, and alpha-fetoprotein were not raised. Due to high suspicion of metastatic nature of the orbital tumor, systemic surveillance with CT was done, which revealed a stage four left RCC with para-aortic nodes, lungs, and bone metastases (Figure 4). A clinical diagnosis of left metastatic orbital tumor secondary to RCC was made. She refused biopsy for histopathological confirmation, surgery, chemotherapy or radiotherapy, and opted for palliative care.

## Discussion

Orbital metastases are rare, comprising 1-13% of all orbital tumors. The majority of orbital metastases occur after a confirmed diagnosis of primary cancer, only 25% present as the first manifestation of metastatic cancers. The most common primary tumors are breast cancer (53%), prostate carcinoma (12%), and lung cancer (8%) [1]. Orbital metastasis from RCC is uncommon (3.2%) [1-2]. In general, extraocular muscles are most frequently involved due to their abundant blood supply, followed by bone marrow space of sphenoid due to the relatively high volume of low-flow blood in it [3]. Breast cancer tends to spread to orbital fat and muscle, while prostate cancer to orbital bone [4]. RCC commonly metastasizes via hematogenous and lymphatic pathways to lungs, intra-abdominal organs, and brain, less commonly to paranasal sinuses, nose, and oral cavity [5]. Metastasis to the eye is rare, involving the uvea most commonly.

More than 90% of orbital metastases occur unilaterally [1]. Predominant clinical features include limited eye movement, diplopia, dystopia, proptosis, decreased vision, ptosis, and palpable mass [6]. Clinically, rapid progression suggests malignancy. Differentiation between a primary and metastatic tumor is difficult clinically, especially when without a known primary cancer or systemic features. Other differential diagnoses include orbital lymphoma, cavernous hemangioma, orbital lymphangioma, lacrimal gland tumor, and rhabdomyosarcoma.

Orbital mass pulsation can be due to transmission of cerebrospinal fluid pulsation through a destructed orbital bone, or tumor intrinsic pulsation secondary to high internal blood flow, for example in tumor of thyroid origin [3]. Other benign causes are carotid-cavernous fistula and cephalocele. Pain is a non-specific symptom that can be caused by mass effect, inflammation, or perineural spread of the tumor. However, the absence of pain does not exclude malignancy or systemic spread.

Imaging with CT or MRI is invaluable in demonstrating the extent of the tumor and narrow down the possible disease entities. The presence of bony erosion is highly suggestive of an aggressive lesion, as opposed to osseous remodeling in a slow-growing lesion [7]. Lytic destruction of the greater wing of the sphenoid is highly suspicious of metastatic disease [3].

In this case, owing to its rapid progression, a malignant tumor was suspected. Location of mass at superotemporal quadrant of orbit was suggestive of lacrimal gland tumor. However, points against it were inferotemporal dystopia and a pulsatile mass. Orbital metastasis was also suspected, but the systemic review showed no related symptoms or signs. Other differential diagnoses were orbital lymphangioma and orbital lymphoma. Orbital CT scan features of an invasive lesion with sphenoid bone destruction lead to an active search of the primary tumor which included breast cancer in view of positive family history, and lung cancer, which is the most common malignancy among smokers.

RCC accounts for 2-3% of all cancers. Risk factors include smoking, obesity, hypertension, and kidney cancer in first-degree relatives [8]. Pertaining to her family history of breast cancer, a literature search showed phosphatase and tensin homolog (PTEN) gene mutation is associated with a high risk of both breast and kidney cancers [9]. Only 6-10% of RCC patients present with a classic triad of flank pain, gross haematuria, and a palpable abdominal mass. Most patients present late with advanced disease. Imaging is the main diagnostic tool [8]. Nephrectomy and metastasectomy are recommended for resectable lesions in metastatic disease, followed by chemotherapy. Radiotherapy is useful in the palliative treatment of bone and brain metastases. The prognosis of metastatic RCC is poor with a five-year survival rate of 8% [10].

## Conclusions

Metastatic RCC can present with non-axial proptosis in the absence of clinical signs or symptoms of a primary tumor. A heterogenous ill-defined extraconal and intraconal orbital mass with lytic destruction of the sphenoid wing should raise the suspicion of a metastatic lesion. A high index of suspicion leading to an active search for the primary tumor may aid in the timely diagnosis and management of patients with asymptomatic primary tumors.
